# Xeno-miRNet: a comprehensive database and analytics platform to explore xeno-miRNAs and their potential targets

**DOI:** 10.7717/peerj.5650

**Published:** 2018-09-28

**Authors:** Yannan Fan, Maria Habib, Jianguo Xia

**Affiliations:** 1 Institute of Parasitology, McGill University, Ste. Anne de Bellevue, Quebec, Canada; 2 Department of Animal Science, McGill University, Ste. Anne de Bellevue, Quebec, Canada; 3 Department of Business Information Technology, University of Jordan, Amman, Jordan

**Keywords:** Xeno-miRNA, Network analysis, miRNA, Cross-species communication, Exosome

## Abstract

Xeno-miRNAs are microRNAs originating from exogenous species detected in host biofluids. A growing number of studies have suggested that many of these xeno-miRNAs may be involved in cross-species interactions and manipulations. To date, hundreds of xeno-miRNAs have been reported in different hosts at various abundance levels. Based on computational predictions, many more miRNAs could be potentially transferred to human circulation system. There is a clear need for bioinformatics resources and tools dedicated to xeno-miRNA annotations and their potential functions. To address this need, we have systematically curated xeno-miRNAs from multiple sources, performed target predictions using well-established algorithms, and developed a user-friendly web-based tool—Xeno-miRNet—to allow researchers to search and explore xeno-miRNAs and their potential targets within different host species. Xeno-miRNet currently contains 1,702 (including both detected and predicted) xeno-miRNAs from 54 species and 98,053 potential gene targets in six hosts. The web application is freely available at http://xeno.mirnet.ca.

## Introduction

MicroRNAs (miRNAs) are ∼22nt non-coding small RNAs mediating post-transcriptional gene silencing by binding to their mRNA targets (Bartel, 2004). Since its discovery, miRNA has been shown to be involved in many biological processes including cell proliferation, cell differentiation, cell migration, disease initiation, and disease progression (Ma, Teruya-Feldstein & Weinberg, 2007; Png et al., 2012; Tay et al., 2008). Recent years have witnessed a growing interest in investigating the potential roles of xeno-miRNAs (miRNAs that have been detected in host biofluids, but originating from different species) in cross-species communications. For instance, studies on helminth infections have found that miRNAs encapsulated in exosomes secreted by those parasites were able to modulate host imMune responses (Buck et al., 2014; Zamanian et al., 2015). It has been shown that miRNAs encoded by Epstein-Barr virus (EBV) could deliver immunomodulatory effect via targeted suppression of key host genes (Xia et al., 2008). Identified in human sera, a plant miRNA was shown to be able to suppress the proliferation of breast cancer cells (Chin et al., 2016). Moreover, a recent study showed that miRNAs secreted by host gut epithelial cells were able to modulate the growth of gut microbiota (Liu et al., 2016). Despite the current controversies regarding xeno-miRNAs from dietary intake (Bagci & Allmer, 2016; Chen, Zen & Zhang, 2013; Dickinson et al., 2013; Kang et al., 2017; Tosar et al., 2014; Witwer & Halushka, 2016), there have been increasing interests to understand the roles of these xeno-miRNAs due to their potentials for translational applications.

Most of the current bioinformatics resources for miRNA studies were developed to help understand functions of miRNAs within the same organisms (Fan et al., 2016; Kozomara & Griffiths-Jones, 2014; Lu et al., 2012; Ru et al., 2014; Vlachos et al., 2015). Available tools for cross-species interactions focused primarily on host-virus interactions (Elefant et al., 2011; Hsu et al., 2007; Kim et al., 2012; Li, Shiau & Lin, 2008; Qureshi et al., 2014; Shao et al., 2015; Veksler-Lublinsky et al., 2010), although the situation has started to change very recently (Mal, Aftabuddin & Kundu, 2018; Zhang, Resende & Cui, 2017; Zheng et al., 2017). Here we introduce Xeno-miRNet, a web-based database and analytics platform that integrates multiple xeno-miRNA resources to support target search, visual exploration and functional analysis. The key features of xeno-miRNet include: (1) a comprehensive collection of experimentally detected and computationally predicted xeno-miRNAs; (2) systematic target predictions integrating two well-established algorithms; and (3) a fully-featured network visual analytics system that allows users to browse, search and visually explore the results in an intuitive manner.

## Materials and Methods

### Xeno-miRNA collection and curation

We performed a comprehensive literature review and manually collected xeno-miRNA entries from these papers and resources (Bernal et al., 2014; Buck et al., 2014; Chen et al., 2011; Cheng et al., 2013; Chin et al., 2016; Fromm et al., 2015; Gottwein, 2012; Guo et al., 2017; Hao et al., 2010; Tritten et al., 2014; Zamanian et al., 2015; Zhang et al., 2012; Zheng et al., 2017; Zhu et al., 2016a; Zhu et al., 2016b). Xeno-miRNet currently contains 453 xeno-miRNAs from 54 species, detected in six host organisms (*H. sapiens, M. musculus, S. scrofa, G. gallus, D. melanogaster,* and *C. elegans*). Based on the pairing information on host and xeno-species, additional 1,249 xeno-miRNAs were predicted to have high potential to be transferred to human circulation according to a recent computational analysis (Shu et al., 2015).

### Xeno-miRNA target prediction

To identify the putative target genes of these miRNAs in the corresponding host organisms, we first downloaded the 3′ UTR sequences of six host organisms from the Ensembl database and the xeno-miRNA sequences from the miRBase (Kozomara & Griffiths-Jones, 2014). We then evaluated the available algorithms including PicTar (Krek et al., 2005), TargetScan (Grimson et al., 2007), miRanda (Betel et al., 2010), microT (Maragkakis et al., 2009), and TarPmiR (Ding, Li & Hu, 2016)*.* For large-scale miRNA target prediction across many different species, the candidate algorithms must be available for local installation, high-performance, and accepting inputs from different species. Using a powerful workstation (128G RAM and 32 CPU cores), we were able to install miRanda and TarPmiR and complete the tasks within a week. The following cutoff values are used: score ≥140 for miRanda and probability ≥0.5 for TarPmiR. Genes in their overlap were selected as potential targets for a given host. The miRNA-target interaction data was stored into an SQLite database (version 3.0) for fast retrieval. Table 1 shows the summary of the xeno-miRNA database.

**Table 1:** The summary statistics for the xeno-miRNet database.

Hosts	Tissue/ sources	Xeno-species	Xeno-miRNAs (detected/ predicted)	Potential targets
Human	18	40	296/625	20,791
Mouse	18	27	83/418	19,430
Pig	4	14	20/116	12,537
Chicken	2	15	23/10	16,459
Fruit fly	6	6	16/44	12,445
*C. elegans*	8	6	15/36	16,391
Total	49	54 (unique)	1702	98,053

### Xeno-miRNet implementation

The web framework was developed based on the JavaServer Faces (JSF) technology using the PrimeFaces (https://www.primefaces.org) component library (version 6.2). As one miRNA can target more than one mRNAs and one mRNA can be targeted by multiple miRNAs, we employed a network visualization approach to allow users to intuitively explore the “multiple-to-multiple” relationships between xeno-miRNAs and their potential gene targets. The JavaScript library *sigma.js* (http://www.sigmajs.org) was used for high-performance network visualization. The functional enrichment analysis was implemented using the R programming language ( R Core Team, 2018). The entire system is deployed on a Google Cloud server with 30GB of RAM and eight virtual CPUs with 2.6 GHz each.

## Results

**Figure 1:**
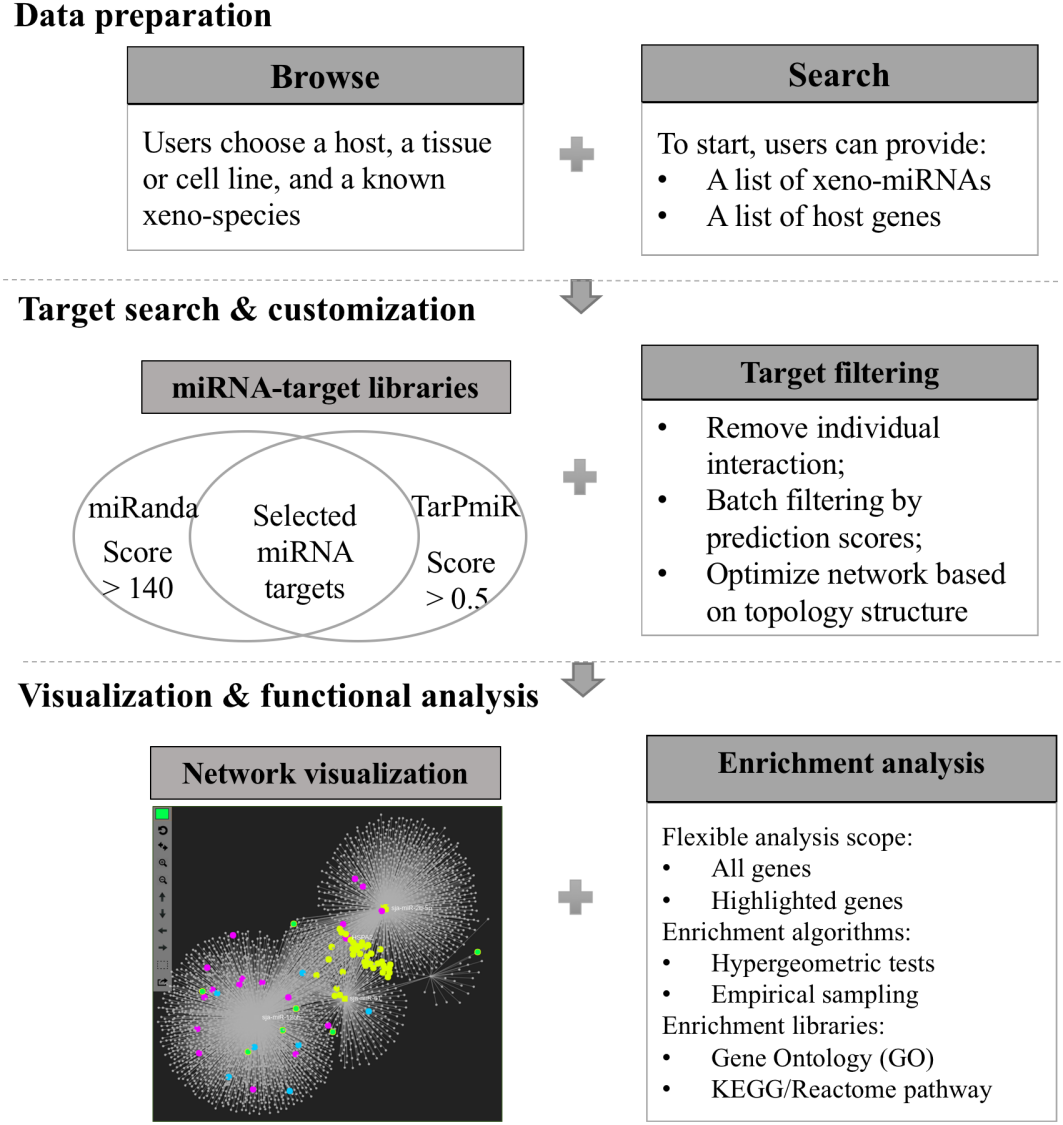
The workflow of Xeno-miRNet. The workflow consists of three steps—data preparation, target search and customization, and the network visual analytics.

Xeno-miRNet has been developed as a database and web-based analytical platform to allow users to query and explore xeno-miRNAs and their potential gene targets in multiple hosts. The website contains a comprehensive list of frequently asked questions (FAQs) and tutorials to help users to start using the tool. There are three major steps—(1) data preparation, (2) target searching and network customization, and (3) network visualization and functional analytics. Figure 1 shows the overall flowchart of Xeno-miRNet. For each step, a variety of options and procedures are provided to help users complete their tasks.

### Data browsing and searching

From the home page, users can start with *Browse* or *Search* by clicking the respective button. To perform *Browse*, users should first specify the host organism. Next, users should select a source and a known xeno-species. For human host, there are 12 different tissue sources and more than 50 xeno-species. To perform *Search,* users should enter a list xeno-miRNAs (miRBase ID or accession number) or a list of host target genes (Ensembl ID, Entrez ID, or official gene symbol). In both modes, the next step is to choose whether to include the predicted xeno-miRNAs. It is important to note that including predicted data may return a large interaction result. To demonstrate the *Search* function, we will use an built-in example “miRNA list1” containing five highly expressed *S. japonicum* exosome miRNAs (*sja-miR-125b, sja-miR-2162-3p, sja-miR-2b-5p, sja-miR-61,* and *sja-miR-10-5p*) (Hao et al., 2010; Zhu et al., 2016a) and explore their potential functions in human host. This list is available as the first example when user click the “Try Examples” when users enter the *Search* page.

### Interaction table refinement

**Figure 2:**
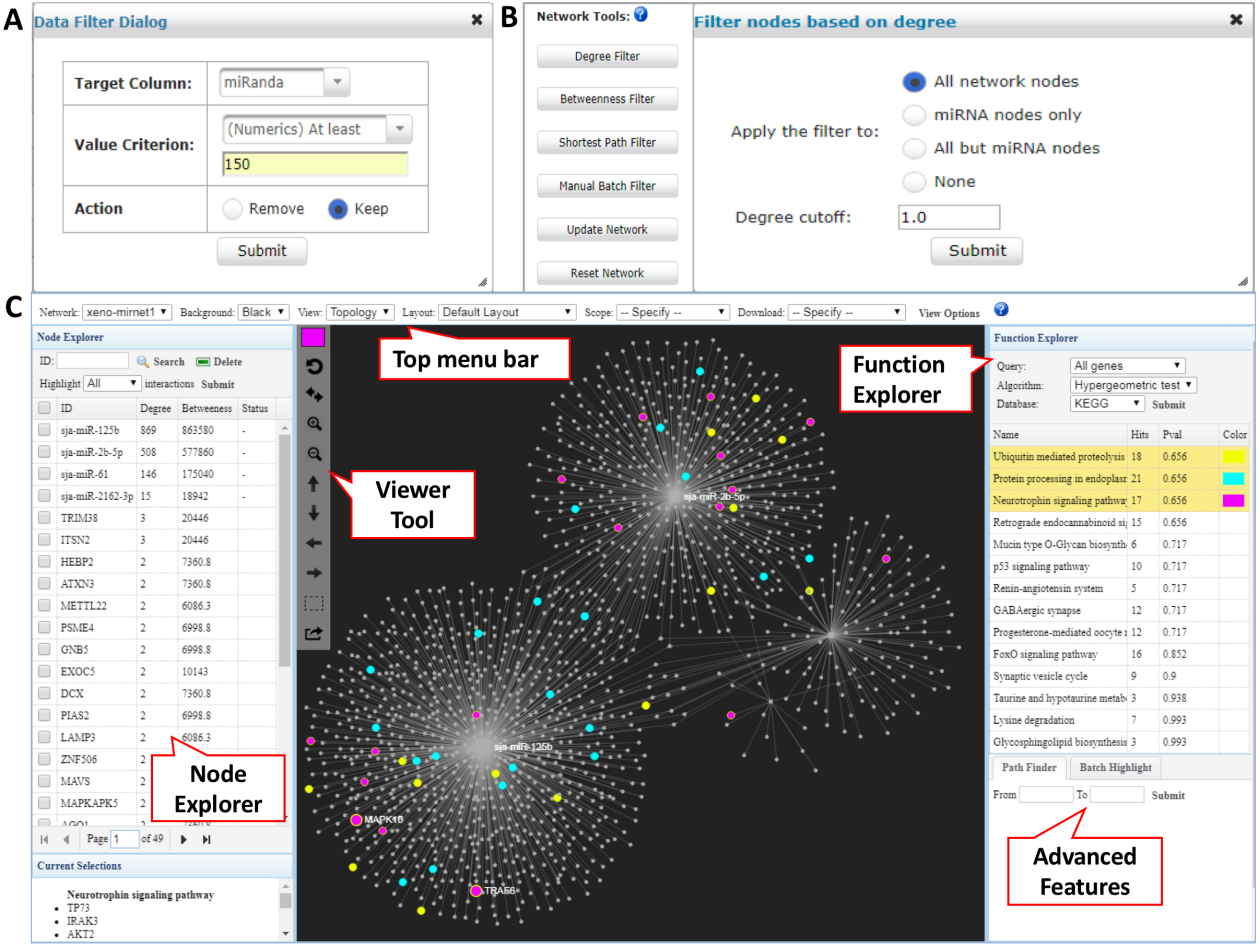
Network customization and visualization. (A) The Data Filter dialog shows how to keep target genes with miRanda scores at least 150. (B) The Network Tools support network refinement. Users can choose corresponding node types and input the cutoff to perform the filtering. (C) The Network Visualization page contains comprehensive features for network analysis and visualization.

In the returned interaction table, each row represents a pair of xeno-miRNA and predicted gene target with hyperlinks to their corresponding databases. The table also provides relevant evidence (RNAseq read counts, miRanda and TarPmiR prediction scores) to allow users to assess the quality of the interactions. The *Data Filter* function allows users to refine the results based on certain matching criteria. For example, users can keep the interactions which miRanda scores higher than 150 by choosing the *Target Column* as *miRanda*, typing in *150* in the frame, and selecting *Keep* (Fig. 2A). Users can save the original interaction result into a CSV file. The filtered result will be used for network construction in the next step.

### Network creation and customization

The network builder page shows a summary table of the generated xeno-miRNA-target interaction network(s), with the number of nodes and edges displayed for each network. A large network (i.e., over 2,000 nodes) often leads to a “hairball” effect in which edges are too densely connected to show any pattern. To overcome this issue, we have implemented the *Network Tools* to allow users to filter nodes according to their topological measures (degree, betweenness, and shortest path) to keep those major hubs while still maintain major connection patterns. The degree of a node is the total number of connections it has to other nodes, and nodes with high degrees are considered important “hubs” in a network. The betweenness value measures the number of the shortest path going through a node, and nodes with high betweenness values are important “connectors” in a network. The shortest path option is for reducing the number of edges within the network by keeping only one shortest path between the hub nodes (Fig. 2B). These functions can work with the previous *Data Filter* to allow users to have fine control over the resulting network for visualization in the next step.

### Network visual exploration

The overview of the network display is shown in Fig. 2C. The network visualization page is composed of four main components—(1) the top tool menu, (2) the *Node Explorer* panel, (3) the central network display panel, and (4) the *Function Explorer* panel. The top tool menu allows users to specify which sub-network to display and to control the overall style of the network. The *Network* option provides a drop-down menu listing all networks that are available to display. Users can specify the currently displayed network and the default is the largest one (“xeno-mirnet1” in Fig. 2C). The *Background* option can be used to switch between black and white background. The *Layout* option allows users to arrange the node positions of the network. The *Scope* option allows users to control the nodes being affected when users manually drag or highlight a single or a group of nodes. The *View Options* allow users to modify the styles for nodes, edges, and highlighting. The *Node Explorer* displays all the nodes in the current network. Nodes are identified by their IDs or names, together with degree and betweenness values. Users can sort the table by clicking a column header. Clicking a node will highlight it within the current network. In addition, user can select multiple miRNAs and then highlight the gene targets shared by them using the *Highlight* function. The central display area is for visual exploration of the network with a vertical toolbar on the left. The color palette located at the top of the toolbar allows users to define the current highlighting color for nodes selection. Users can perform zooming, highlighting, drag-and-drop, or extracting the highlighted nodes using a mouse movement in combination with functions in the toolbar. The button with a dotted rectangle icon allows users to manually select a group of nodes. After clicking this icon, users can use mouse to select a group of nodes of interest for further functional analysis. The *Function Explorer* allows users to perform enrichment analysis to identify important functions defined by gene ontology (GO), KEGG or Reactome pathways. Two algorithms have been implemented - the *hypergeometric tests* and the *empirical sampling* as recently proposed by Bleazard, Lamb & Griffiths-Jones (2015) for more robust miRNA target enrichment analysis. The result is a list of functions ranked by their *p*-value. Users can highlight the nodes involving in the pathway by simply clicking on the function name. Figure 2C shows the result after performing the KEGG pathway analysis to the targets from the *S. japonicum* exosome miRNAs. The “Protein processing in endoplasmic reticulum” (highlighted in blue) and “Endocytosis” (highlighted with purple) were identified as significant pathways. When a network is too complex, users can extract a module or sub-network containing only the nodes of interest by using the *Extract* button on the central display toolbar (the bottom one). The extracted module will be listed as “module1” in the *Network* option on the top toolbar and the sub-network will be displayed in the center viewer. Users can perform further customization for the sub-network.

## Discussion

To address the growing bioinformatics needs for xeno-miRNA research, several tools have been developed recently. For instance, Exo-miRExplorer is a database curating exogenous miRNAs detected from high-throughput small RNA sequencing experiments (Zheng et al., 2017); miRDis is a web service that supports discovery and annotation of exogenous miRNAs from small RNA sequencing data (Zhang, Resende & Cui, 2017); IIKmTA is a new tool that aims to support both inter- and intra- kingdom miRNA-target analysis (Mal, Aftabuddin & Kundu, 2018). Table 2 compares the key features between Xeno-miRNet and these recent tools. Based on the comparison, it is evident that Xeno-miRNet complements other tools by providing comprehensive support for functional analysis and network-based visual exploration. It is important to note that Xeno-miRNet currently focuses on the six model organisms with extensive literature support. We intend to gradually expand the range of host organisms based on user feedback and available data.

**Table 2:** Comparison with other tools available for xeno-miRNA analysis. The “ +” and “ −” are used to indicate if features are present or not. More “ +” indicate better support.

**Tools**	**Xeno-miRNet**	**Exo-miRExplorer**	**IIKmTA**	**miRDis**
**Hosts #**	6	13	116	6
**Xeno-species #**	54	64	109	8
**xeno-miRNA sources**				
Experimental detected	+	+	−	+
Predicted	+	−	+	−
**Input data**				
miRNAs	+	+	+	+
Targets	+	−	−	**-**
Expression data	−	−	−	**+**
**Result presentation**				
Interaction table	+	−	+	−
Network visualization	+++	−	−	−
**Enrichment analysis**				
Hypergeometric tests	+	−	−	−
Empirical sampling	+	−	−	−

**Notes.**

Xeno-miRNet: http://xeno.mirnet.ca.

Exo-miRExplorer: http://rna.sysu.edu.cn/exomiRDB/.

IIKmTA: http://www.bioinformatics.org/iikmta/.

miRDis: http://sbbi.unl.edu/miRDis/index.php.

## Conclusions

A growing number of studies have suggested xeno-miRNAs as an important means in cross-species interactions and communications. In this manuscript, we introduced Xeno-miRNet, a user-friendly web-based tool developed through comprehensive curation of xeno-miRNAs and systematic predictions of their potential gene targets in multiple hosts. Xeno-miRNet offers a platform to allow researchers to intuitively explore both detected and potential xeno-miRNAs within the context of miRNA-target gene interaction networks to obtain functional insights. It is expected that Xeno-miRNet will help researchers to generate and to refine hypotheses for more targeted experimental studies to accelerate scientific discoveries and their potential translations.

## Additional Information and Declarations

### Data Availability

The following information was supplied regarding data availability:
http://xeno.mirnet.ca: Integrates data, algorithms and visualizations.
